# Host obesity impacts genetic variation in influenza A viral populations

**DOI:** 10.1128/jvi.01778-23

**Published:** 2024-05-24

**Authors:** Marissa Knoll, Rebekah Honce, Victoria Meliopoulos, Ernesto Alejandro Segredo-Otero, Katherine E.E. Johnson, Stacey Schultz-Cherry, Elodie Ghedin, David Gresham

**Affiliations:** 1 Department of Biology, Center for Genomics and Systems Biology, New York University, New York, New York, USA; 2 Department of Infectious Diseases, St. Jude Children’s Research Hospital, Memphis, Tennessee, USA; 3 Systems Genomics Section, Laboratory of Parasitic Diseases, NIAID, NIH, Bethesda, Maryland, USA; Emory University School of Medicine, Atlanta, Georgia, USA

**Keywords:** obesity, influenza, ferret, genetic diversity

## Abstract

Obesity is well established as a risk factor for many noncommunicable diseases; however, its consequences for infectious disease are poorly understood. Here, we investigated the impact of host obesity on influenza A virus (IAV) genetic variation using a diet-induced obesity ferret model and the A/Hong Kong/1073/1999 (H9N2) strain. Using a co-caging study design, we investigated the maintenance, generation, and transmission of intrahost IAV genetic variation by sequencing viral genomic RNA obtained from nasal wash samples over multiple days of infection. We found evidence for an enhanced role of positive selection acting on *de novo* mutations in obese hosts that led to nonsynonymous changes that rose to high frequency. In addition, we identified numerous cases of mutations throughout the genome that were specific to obese hosts and that were preserved during transmission between hosts. Despite detection of obese-specific variants, the overall viral genetic diversity did not differ significantly between obese and lean hosts. This is likely due to the high supply rate of *de novo* variation and common evolutionary adaptations to the ferret host regardless of obesity status, which we show are mediated by variation in the hemagglutinin and polymerase genes (PB2 and PB1). We also identified defective viral genomes (DVGs) that were found uniquely in either obese or lean hosts, but the overall DVG diversity and dynamics did not differ between the two groups. Our study suggests that obesity may result in a unique selective environment impacting intrahost IAV evolution, highlighting the need for additional genetic and functional studies to confirm these effects.

**IMPORTANCE:**

Obesity is a chronic health condition characterized by excess adiposity leading to a systemic increase in inflammation and dysregulation of metabolic hormones and immune cell populations. Influenza A virus (IAV) is a highly infectious pathogen responsible for seasonal and pandemic influenza. Host risk factors, including compromised immunity and pre-existing health conditions, can contribute to increased infection susceptibility and disease severity. During viral replication in a host, the negative-sense single-stranded RNA genome of IAV accumulates genetic diversity that may have important consequences for viral evolution and transmission. Our study provides the first insight into the consequences of host obesity on viral genetic diversity and adaptation, suggesting that host factors associated with obesity alter the selective environment experienced by a viral population, thereby impacting the spectrum of genetic variation.

## INTRODUCTION

Obesity is an increasing public health concern as the number of people who are considered obese has nearly tripled since 1975, with over 600 million adults considered overweight in 2015 (1). Although primary health risks associated with obesity include cardiac and metabolic syndromes, obesity is also known to alter immune function through the endocrine action of adipocytes, which are both enlarged and more numerous in obesity (2, 3). Adipocytes release immune related proteins, including leptin, which has been shown to upregulate the production of pro-inflammatory cytokines (4, 5). In addition, adipocytes secrete higher levels of immune proteins both locally, including TNF-α, and systemically, including C-reactive protein, interleukin 6 (IL-6), and serum amyloid A (SAA) (3, 6). Obesity is also linked to dysregulation of immune cells, such as reduced lymphocyte proliferation and elevated leukocyte and lymphocyte counts (6, 7), putatively due to the increased systemic inflammation and dysregulated metabolic hormones. Obesity has been shown to exacerbate noncommunicable disease, such as cardiovascular disease, metabolic syndromes, cancer, and infectious diseases (8–10). The link between host obesity and infectious diseases is especially interesting as changes in immune function could contribute significantly to infection dynamics and disease severity.

Influenza A virus (IAV), a single stranded, negative-sense segmented RNA virus, is a common respiratory pathogen that causes infection of the upper and, in severe cases, the lower respiratory tracts (11). Obesity is implicated in more severe disease and increased mortality in IAV-infected individuals (12–15). However, the underlying mechanisms have not been fully elucidated. Case studies and population data have shown increased IAV shedding, indicative of higher viral loads, in obese patients (16, 17). In addition, obese patients have lower type I interferon production and delayed antiviral responses (18). These factors may impair the ability of an obese host to suppress viral replication, resulting in delays in viral clearance and increases in the length of illness. Animal studies provide additional evidence for these effects as high-calorie diets led to altered metabolic and immunological profiles and increased mortality in response to IAV infection (19–21).

As with all RNA viruses, the error-prone nature of the RNA-dependent RNA polymerase in IAV can lead to significant within-host genetic diversity of the viral population (22). This genetic diversity can take many forms including single-nucleotide variants (SNVs) and defective viral genomes (DVGs), which we define here as large internal deletions within segments that retain conserved 3′ and 5′ ends (23). The genetically diverse population within a host experiences selective pressure from the host environment, leading to allele frequency changes of SNVs, especially those in the genes that code for the antigenically variable surface proteins hemagglutinin (HA) and neuraminidase (NA) (22). By contrast, DVGs are predominantly found in the polymerase gene segments (PB2, PB1, and PA) (23). The extent to which the altered metabolic and immune status of obese hosts impacts the accumulation and selection of IAV genetic variation has not previously been investigated.

To study the accumulation of genetic variation in IAV populations over the course of infection, we developed a diet-induced obesity model in ferrets (24). The use of ferrets as a model for human IAV infections is well established and has a number of advantages, including their susceptibility to, and transmissibility of, human-relevant strains without extensive initial host adaptation (25–28). Thus, using an obese ferret model allows us to examine IAV infections in a physiologically relevant context for human obesity.

Human IAV infections are most frequently caused by H1N1 and H3N2 subtypes; however, many other subtypes can cause disease and thus are of major health concern. H9N2 is a low-pathogenicity avian-like influenza virus with reservoirs in wild bird populations and domestic poultry, which has led to occasional human infections (29). Although there is no current evidence for human-to-human transmission of H9N2, it remains a virus of pandemic concern by the CDC (30). Ferret models have been used to investigate H9N2 infectivity and transmissibility (31). However, the effect of obesity on the genetic diversity in the viral population during H9N2 infection has not previously been studied.

To investigate the effect of host obesity on genetic diversity, we infected neutered male obese ferrets and lean ferrets with the A/Hong Kong/1073/1999 (H9N2) strain. Using a co-caging study design, we investigated the generation and transmission of intrahost genetic diversity in obese and lean animals. We assayed genetic diversity in the viral population through optimized experimental and computational workflows to quantify intrahost SNV (32) and DVG (https://github.com/GhedinSGS/DiVRGE) diversity from Illumina sequencing data across multiple days of infection and transmission pairs. We find evidence of positive selection in the form of nonsynonymous SNVs that increased to high frequencies in obese contact ferrets independent of transmission and multiple recurrent low-frequency variants that were unique to obese ferrets. In addition, we find evidence that adaptation to the ferret host was mediated by variation in HA and polymerase genes (PB2 and PB1) and was independent of diet. Despite these differences, SNV and DVG diversity did not differ in obese hosts compared with lean hosts. Our study suggests that host factors associated with obesity alter the selective environment experienced by the viral population, resulting in a unique class of obese-specific genetic variants, although further work is needed to confirm that the differential selection is due specifically to host obesity.

## RESULTS

### Study design

**Fig 1:**
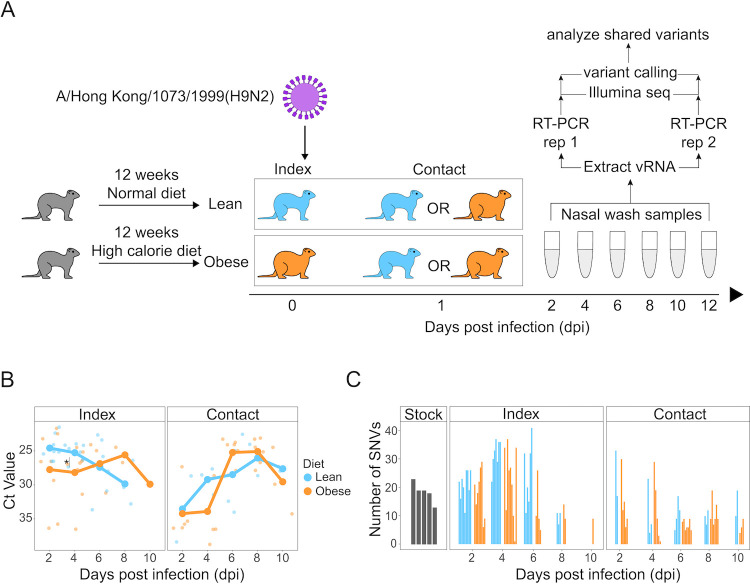
Infection of lean and obese ferrets with A/Hong Kong/1073/1999 (H9N2) virus. (**A**) Schema of experimental design: Influenza-naive neutered male young adult ferrets were fed a lean or obese diet for 12 weeks prior to infection with A/Hong Kong/1073/1999 (H9N2) influenza virus. Index ferrets were inoculated intranasally with 10^6^ TCID_50_ on day 0. An index ferret was co-caged with a contact ferret at 1 dpi. All possible combinations of lean and obese index and contact ferrets were tested. At 2-day increments from 2 to 12 dpi, nasal wash samples were collected and viral RNA was isolated, reverse transcribed, amplified, and sequenced in duplicate. (**B**) Viral load was quantified using reverse transcription-PCR (RT-PCR) analysis of the M gene and estimated using the cycle threshold (Ct) method for all samples passing sequencing quality thresholds. Small points indicate values for individual samples. Large points connected by lines are average values of samples for each diet group. Facets indicate infection route and color indicates diet. *P < 0.05 using two-tailed Student’s *t*-test. (**C**) Number of SNVs identified per sample (*n* = 81) at each time point. The five different stock values correspond to the five inocula for the five different cohorts used in our study. Individual samples from ferret nasal washes are sorted by dpi.

We investigated the effect of host obesity on IAV genetic diversity during infection using a ferret obesity model. Briefly, neutered male ferrets were randomly assigned to the lean or obese group and reared on the appropriate diet for 12 weeks (33) (Fig. 1A). Infection experiments were performed in five cohorts over the course of roughly 2.5 years (Fall 2017, Winter 2017, Summer 2018, Spring 2019, and Spring 2020) (Table S1). To generate a stock of virus, the H9N2 strain A/Hong Kong/1073/1999 was propagated in 9-day-old embryonated chicken eggs and then stored. One aliquot per cohort was thawed and diluted in phosphate-buffered saline (PBS) for use as the inoculum in our experiments. Index ferrets were infected intranasally with 10^6^ tissue culture infectious dose-50 (TCID_50_) of the A/Hong Kong/1073/1999 (H9N2) inoculum. On the first day post-infection (dpi), an influenza-naive contact ferret was introduced to the same cage as a single index ferret. Our experimental design included all pairwise combinations of index and contact ferrets, namely, lean index and lean contact, lean index and obese contact, obese index and lean contact, and obese index and obese contact (Table S2). Index and contact ferrets remained co-caged until the end of the experiment at 12 dpi. Viral RNA was extracted from nasal wash samples collected every second day from 2 dpi to 12 dpi from both index and contact ferrets. All RNA samples were reverse transcribed, PCR amplified, and sequenced in duplicate. RNA from the viral inoculum was also extracted and sequenced to define pre-existing diversity.

We studied a total of 28 index (15 obese and 13 lean) and 20 contact (12 obese and 8 lean) ferrets over the course of the experiment acquiring a total of 180 nasal wash samples from 48 ferrets over 2–12 dpi. Each ferret was assigned a unique identification code (Table S1). After applying sequencing quality thresholds (see Materials and Methods), our data set comprised 37 ferrets for which at least one sample was suitable for SNV analysis resulting in a total of 81 successfully sequenced samples. In general, failure to acquire sequence data was due to either unsuccessful infection of the index ferret, unsuccessful transmission, or clearance of the infection in infected ferrets at later time points in the experiment. All sequenced samples were acquired from 2 to 10 dpi, and no sequencing data were successfully obtained from samples at 12 dpi suggesting that the infection had been cleared in all individuals by this point. Our sequencing data set includes 11 successful transmission pairs, for which both index and contact ferrets had at least one successfully sequenced sample (Table 1). The average sequence coverage across all eight genome segments exceeded 1,000-fold enabling sensitive detection of low-frequency variants (Fig. S1A).

**TABLE 1:** Distribution of the 37 unique ferrets with successful IAV sequencing^*a*^

Index diet	Transmission events	Contact diet
Obese (*n* = 10)	Obese to obese (*n* = 7)	Obese (*n* = 7)
Obese (*n* = 2)	Obese to lean (*n* = 0)	Lean (*n* = 1)
Lean (*n* = 4)	Lean to obese (*n* = 2)	Obese (*n* = 3)
Lean (*n* = 7)	Lean to lean (*n* = 2)	Lean (*n* = 3)

^*a*^ The number of obese and lean index and contact ferrets for which high-quality sequencing data were acquired. A successful transmission event is defined by the acquisition of sequence data for both an index ferret and its transmission partner. In some cases, data were obtained for a contact ferret but not for the corresponding index ferret and therefore were not considered a positive transmission event.

To quantify viral load, we performed quantitative RT-PCR of the M genomic segment and determined the cycle theshold (Ct) value for all 81 samples that passed quality control filters (Fig. 1B). Lower Ct values indicate more viral RNA material. We found that the overall trend in Ct does not differ significantly between obese and lean index and contact ferrets with the exception of 4 dpi in the index ferrets. This suggests that the total number of viral genomes did not differ between lean and obese hosts.

We performed single-nucleotide variant detection on all 81 samples using a 1% allele frequency cutoff, requiring that variants were identified in both technical replicates (32). Inoculum samples used to infect index ferrets are expected to be genetically heterogeneous as a result of amplification in cell culture. Indeed, we identified an average of 18.4 SNVs in the five different inoculum aliquots, with 86% of the SNVs found in at least four of five aliquots (Fig. 1C Table S3). Variant calling for all ferret samples was performed relative to the consensus sequence of the inoculum for each cohort. Ferret samples contained an average of 18 SNVs (Fig. 1C). We find that samples from the contact ferrets were more genetically diverged from the inoculum than samples from the index ferrets (Fig. S1B). Viral Ct values were negatively correlated with the number of SNVs in index ferrets, consistent with larger population sizes (i.e., more viral RNA genomes resulting in a lower Ct value) exhibiting greater genetic diversity (Fig. S1C). Interestingly, this was not the case for contact ferrets, as Ct values were not correlated with the number of variants. When comparing similar viral abundances, we observe systematically reduced diversity in contact ferrets compared with index ferrets likely reflecting differences related to direct and transmitted infection (Fig. S1C).

### Unique consensus changes are found in obese hosts

As obesity has been shown to lead to significant differences in gene expression and immune function, we hypothesized that different host selection pressure in obese ferrets may result in diet-specific adaptation. Therefore, we identified SNVs that were unique to the viral populations in either the obese or lean diet groups. We distinguish between consensus changes, in which 50% or more of sequence reads at a specific site contains a different nucleotide than the viral inoculum, and minor variants, in which fewer than 50% of sequence reads at a specific site contains a different nucleotide than the viral stock. Consensus changes represent the most relevant variants in terms of evidence for positive selection, due to their high frequency.

We first investigated consensus changes as the high frequency of these SNVs could be a result of positive selection. There were 13 consensus changes identified in 8 of the 37 ferrets (9 in obese ferrets and 4 in lean ferrets) (Table 2). Among these 8 ferrets, 4 of them had more than one consensus change. Surprisingly, nearly all (12 of 13) of the consensus changes were found in contact ferrets. About half of these appear to be *de novo*, and not transmitted, variants as they were not present in the inoculum or their index transmission partner at any detectable frequency (Table 2). The consensus changes that were first found as minor variants in the inoculum, and in the index ferrets, did not rise above 50% frequency until after their transmission to contact ferrets. Two of these variants, L628M on the PB1 segment and F9F on the PA segment, increased to high frequencies in two unrelated contact ferrets. Of the variants that were first found in the stock but rose to high frequency during the infection, all rose to high frequency in obese ferrets, with the exception of the synonymous PA F9F variant. This suggests that whereas these variants were purged or maintained at low frequencies in lean ferrets, they persisted in the obese ferrets, consistent with differential selective pressures in the context of obesity.

**TABLE 2:** Sequence consensus changes identified in infected ferrets^*a*^

Segment	Mutation type	Nucleotide change	Amino acid change	Infection route	Diet	Ferret ID	Source
**PB1**	**Nonsynonymous**	**C1882A**	**L628M**	**Contact**	**Obese**	**1409**	**Stock**
NP	Nonsynonymous	C326T	T109I	Contact	Obese	1409	Stock
PB1	Nonsynonymous	T965C	I322T	Contact	Obese	1980	Stock
**PA**	**Synonymous**	**C27T**	**F9F**	**Contact**	**Obese**	**2231**	**Stock**
PB2	Synonymous	C639A	R213R	Contact	Obese	2232	Stock
**PB1**	**Nonsynonymous**	**C1882A**	**L628M**	**Contact**	**Obese**	**2232**	**Stock**
**PA**	**Synonymous**	**C27T**	**F9F**	**Index**	**Lean**	**2254**	**Stock**
HA	Nonsynonymous	C284A	S95Y	Contact	Obese	1794	*De Novo*
PB2	Nonsynonymous	C334T	P112S	Contact	Lean	1797	*De Novo*
PA	Synonymous	A1483C	R495R	Contact	Lean	1986	*De Novo*
NS	Nonsynonymous	C652T	Q218X	Contact	Lean	1986	*De Novo*
NS	Synonymous	C267T	Y89Y	Contact	Obese	2231	*De Novo*
HA	Synonymous	A747G	Q249Q	Contact	Obese	2232	*De Novo*

^*a*^ The 13 consensus changes (allele frequency greater than or equal to 50%) at the nucleotide and amino acid level found in eight ferrets. Recurrent consensus changes are indicated in bold. Source column indicates whether variants were found as minor variants in the stock or arose *de novo* in the ferret

**Fig 2:**
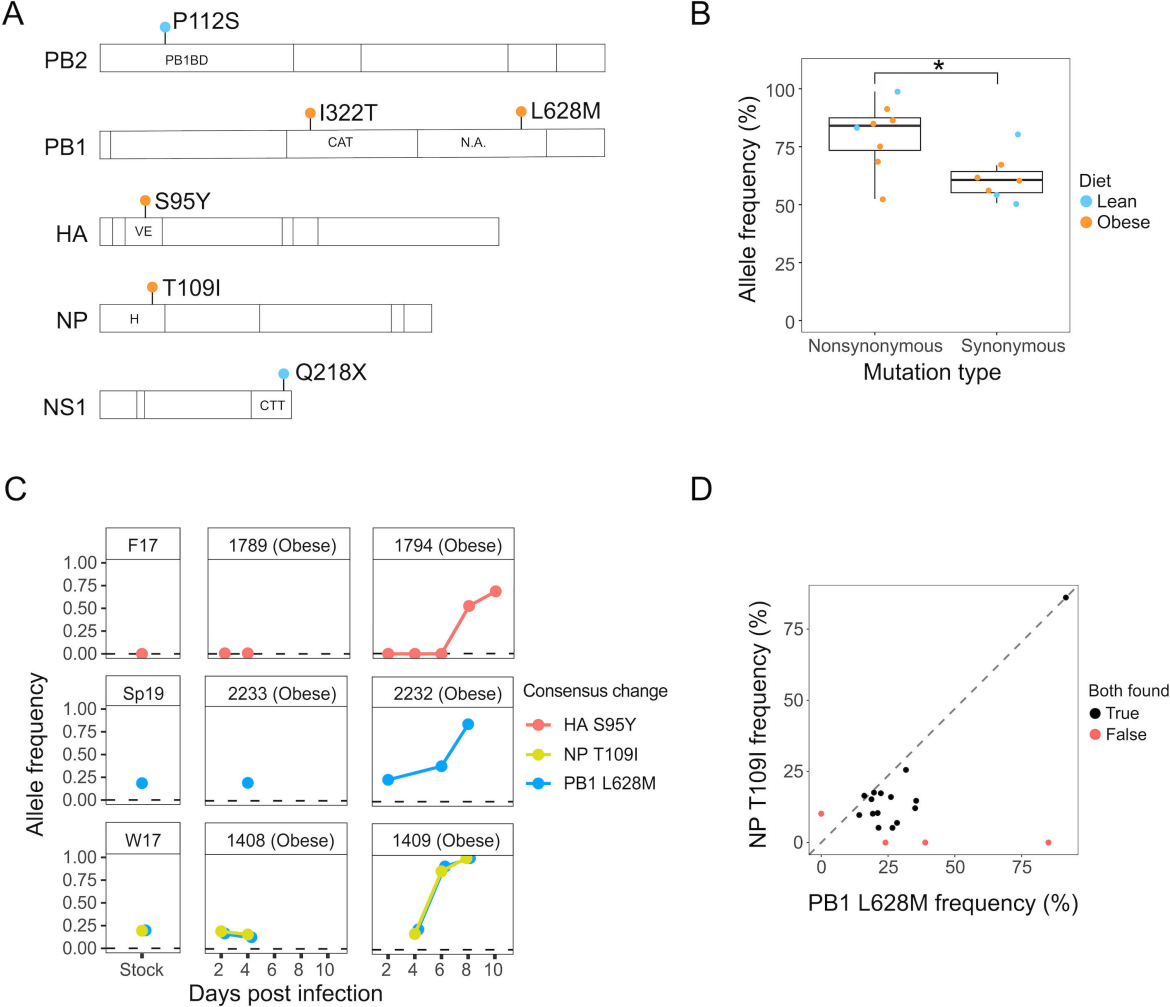
Properties of consensus sequence changes in infected ferrets. (**A**) Schematic of protein domains containing nonsynonymous consensus changes. PB1BD, PB1 binding domain; CAT, catalytic domain; N.A., not annotated; VE, vestigial esterase domain; H, head domain; CTT, C terminal tail. The predicted amino acid change of the consensus variants is labeled. The Q218X nonsense consensus change on the NS segment is specific to the NS1 splice form. It is also predicted to result in a synonymous (N60N) change in NS2, which is not included in the figure for clarity. (**B**) Allele frequencies of nonsynonymous and synonymous consensus changes. Color indicates the diet of the ferret. *P < 0.05 using two-tailed Student’s *t*-test. (**C**) Allele frequencies of consensus changes that increased over sequential days within a ferret. Points at AF = 0 indicate a sample where there was high-quality sequencing data but the variant was not detected. (**D**) Frequency of L628M and T109I mutations at each timepoint when they co-occurred (*n* = 15, black points) and when they did not co-occur (*n* = 4, red points) in a total of six ferrets. Dashed line indicates equal allele frequencies for the two variants.

About half (7 of 13) of the consensus changes were nonsynonymous and were located in the PB2, PB1, HA, NP, and NS segments (Fig. 2A). Nonsynonymous consensus changes tended to be found at higher allele frequencies than synonymous consensus changes (Fig. 2B). Three of the consensus changes, L628M, T109I, and HA S95Y, showed an increase in allele frequency across multiple days, consistent with positive selection (Fig. 2C). All three of the mutations were found in an obese to obese transmission pair. Viral populations expand rapidly during the initial phases of infection in the contact ferrets, peaking by 6 dpi (Fig. 1B). As the variants found in ferret 1794 and 2232 do not reach high frequency until 8 dpi (Fig. 2C), it is unlikely that a simple founder effect explains the increases in allele frequency. Interestingly, the L628M and T109I mutations, which were identified in the inocula, co-occur as minor variants with similar frequencies in 15 different samples from 6 additional ferrets (Fig. 2D), 5 of which are lean (ferrets 1801, 1973, 1986, 2253, and 2254), and one of which is obese (ferret 1789). However, these variants are only found at high frequency in obese contact ferrets, suggesting that they may be under stronger selection in the obese host environment.

In contrast, none of the consensus changes found in the lean ferrets exhibit the same characteristics as those found in obese ferrets. We identified four consensus changes found in lean ferrets: PA F9F, PB2 P112S, PA R495R, and NS Q218X (Table 2). Two of these, PA F9F and PA R495R, are synonymous changes. NS Q218X is found in ferret 1986, for which only one time point led to successful sequencing, and therefore is inconclusive in terms of its dynamics. PB2 P112S is found at high frequency over multiple time points but was already at extremely high frequency by 4 dpi, despite not being detected at 2 dpi (Table S4). Therefore, this sudden rise to extremely high frequency may be the result of random increases due to a founder effect as the viral population rapidly expanded early during infection.

### Highly recurrent minor variants mediate adaptation to the host

In addition to consensus sequence changes, there were a large number of minor variants (*n* = 567) that were not detected in the stock but arose *de novo* within a ferret (Table S4). We initially focused on the *de novo* minor variants that were shared between two or more ferrets, of which we found 100. Most of these were only shared between two ferrets, but we found variants that were shared with up to 22 ferrets. Using the number of variants found within each ferret, we tested whether the number of shared variants could be explained by neutral evolution alone. To do so, we simulated 37 populations of viral genomes containing randomly generated variants with the same average number per ferret as our data set (mean = 12.5) and determined the number of shared variants between populations. We find that sharing 100 of the 567 *de novo* variants is highly unlikely given neutral evolution (chi squared test, *P* value < 2.2*e*^−16^) and is consistent with recurrent generation and selection of variants.

**Fig 3:**
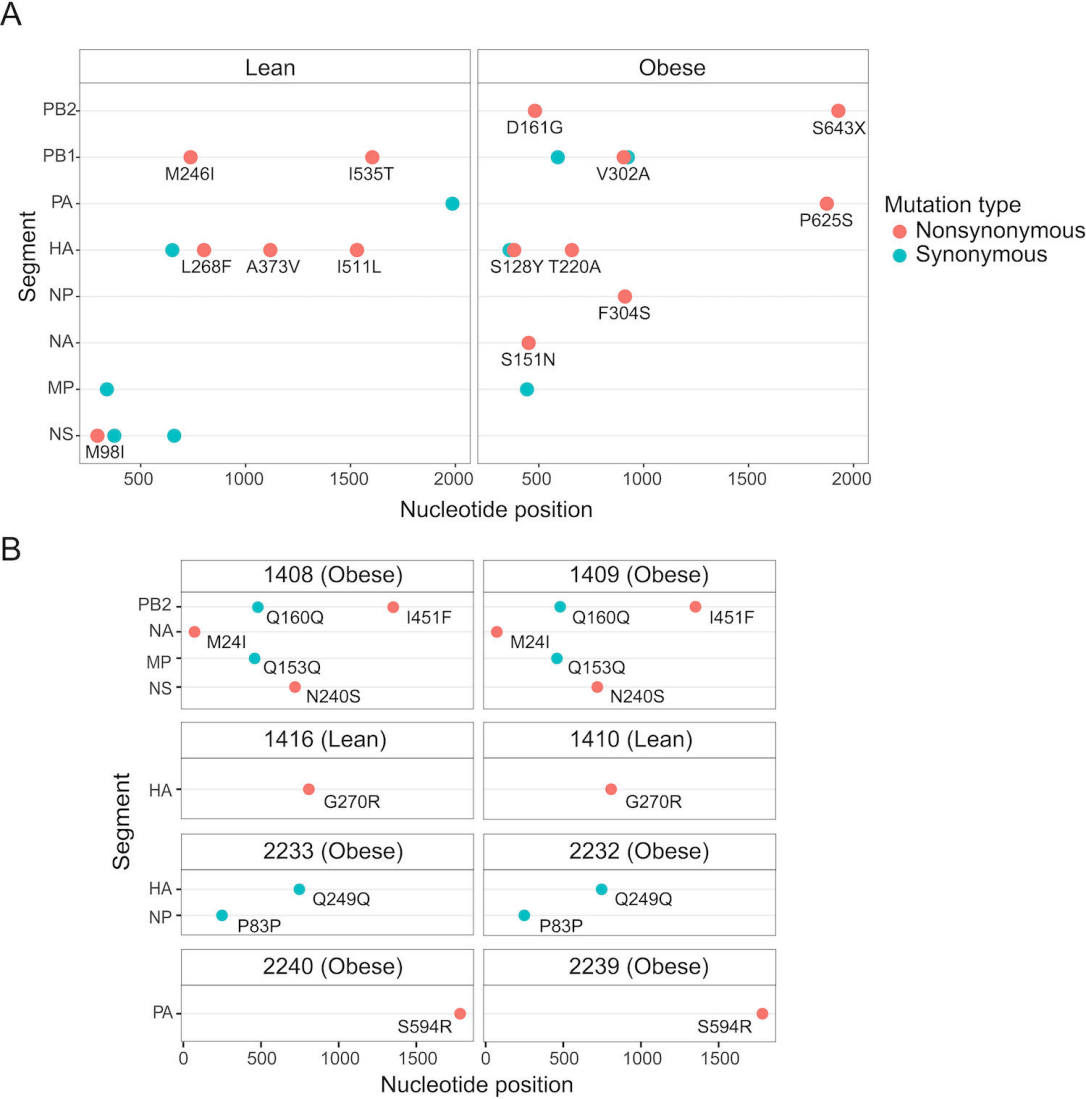
Recurrent and transmitted diet-specific minor variants. (**A**) Minor variants that were found in two ferrets within the same diet group recurrently independent of transmission. Facets indicate diet group. (**B**) Minor variants that were found in the same diet group likely due to transmission. Facets indicate the ferret number and diet of the animals in the transmission pair. In both panels, the color indicates whether the variant is synonymous or nonsynonymous and the nucleotide position and the corresponding amino acid change are indicated.

We then analyzed the distribution of *de novo* minor variants that were shared exclusively within a diet group (i.e., only found within obese ferrets or only found within lean ferrets), as these may be the result of diet-specific adaptation. We found 12 obese-specific variants and 12 lean-specific variants, leaving a total of 67 variants that were shared between ferrets of different diet groups (Fig. 3A). We performed a permutation test to assess the significance of this distribution, based on the number of shared variants (*n* = 100) and how often they were shared (2–22 ferrets). For this analysis, we removed any minor variants that were found in both the index and contact ferret of a single transmission pair, as they were likely to be shared due to transmission, rather than recurrence. We found that variants were not shared exclusively within a diet group more often than predicted by a random distribution (*P* value = 0.5596), even when only considering nonsynonymous variants (*P* value = 0.9369). Shared diet-specific variants also appeared transiently, as we identified only one case in which a variant was present in two sequential time points within an individual (Fig. S2B). However, there were also several cases in which minor variants were transmitted, indicating that these variants can persist through the tight bottleneck that occurs during between-host transmissions (Fig. 3B).

**Fig 4:**
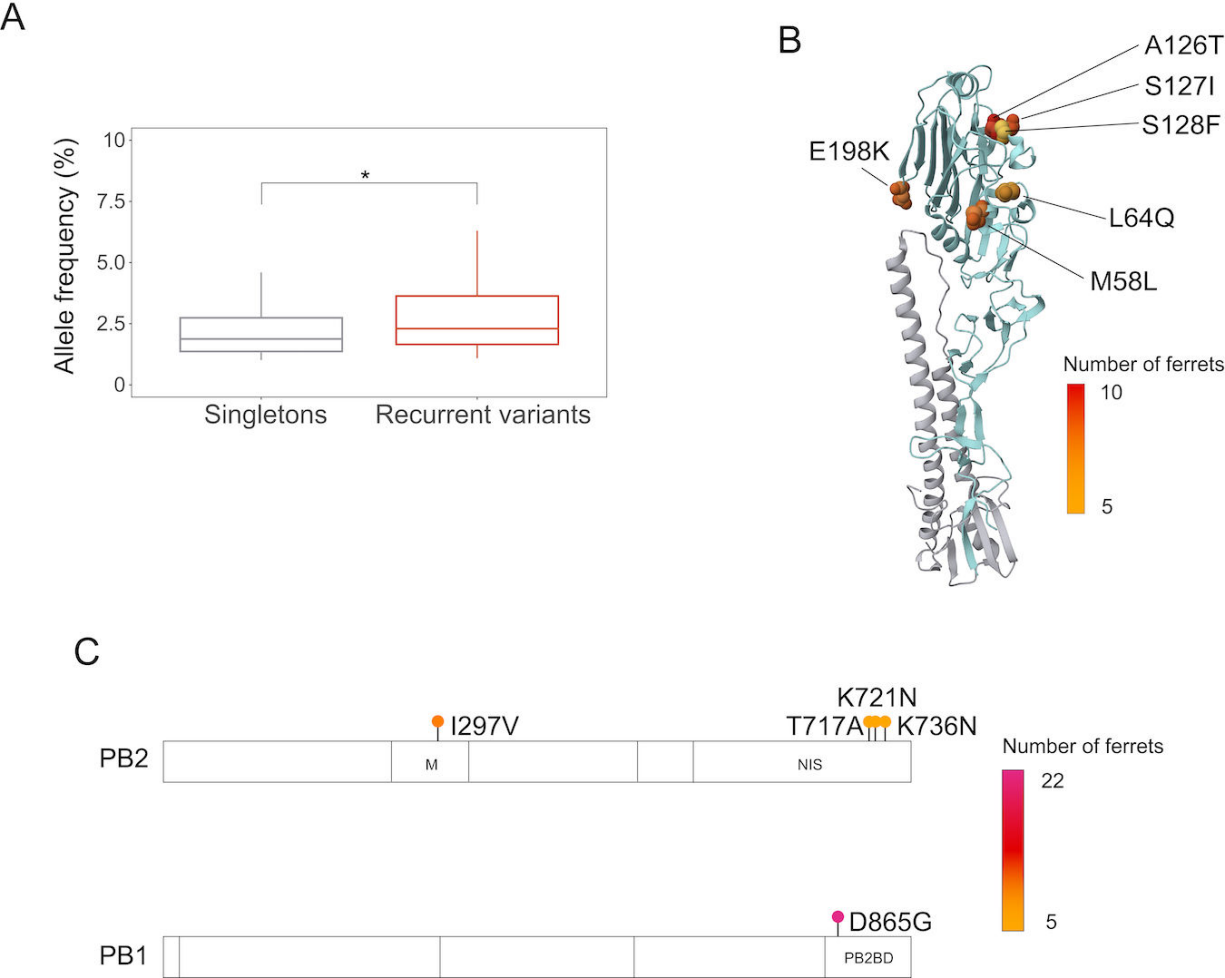
*De novo* nonsynonymous minor variants identified in multiple ferrets independent of diet. (**A**) Allele frequencies of *de novo* minor variants that were present in both obese and lean ferret hosts compared with *de novo* variants that were present in only one ferret. *P < 0.05 using two-tailed Student’s *t*-test. (**B**) Location of shared *de novo* nonsynonymous minor variants mapped onto the 3D structure of the HA protein. Labels denote the amino acid change. Color represents the number of ferrets in which the variant was found. The HA structure of A/swine/Hong Kong/9/98 is shown in its cleaved form (34). (**C**) Position of shared *de novo* nonsynonymous minor variants in PB2 and PB1 segments. Labels denote the corresponding amino acid change. Color represents the number of ferrets in which the variant was found. M, middle; NIS, nuclear import signal; PB2BD, PB2 binding domain.

Recurrent generation of *de novo* variation and its positive selection is rare and therefore difficult to detect. However, the large number of ferrets in our study and the use of an avian-like influenza virus in a mammalian system increased our power to detect these rare events. Recurrent variants were found at higher allele frequencies than minor variants that appear only in one individual (Fig. 4A). We identified a cluster of six highly recurrent variants in the HA1 domain of the HA protein, which contains residues important for binding to host cells (Fig. 4B). Additionally, we find a set of highly recurrent variants in the PB2 and PB1 segments, which encode components of the viral polymerase (Fig. 4C). Of particular interest is the nonsynonymous SNV in the PB1 segment, resulting in an aspartic acid (D) to glycine (G) change at amino acid position 685, which was present at low frequency (1%–5%) in 22 out of 37 ferrets. These highly recurrent *de novo* nonsynonymous variants that occur in both diet groups likely reflect adaptation to the ferret host, rather than host diet-specific adaptations.

### Dynamics of viral diversity over the course of infection

We next sought to quantify the dynamics of viral genetic diversity over the course of infection. Index ferrets were best suited for addressing this question as each individual was infected with similar initial diversity as a result of pre-existing genetic variation in the inoculum and infection with a large inoculum (10^6^ TCID_50_). By contrast, the diversity in contact ferrets depended on the variation in the index ferret and the extent to which it was transmitted, which varied greatly between individuals. In our experimental design, variants present in the inoculum were considered standing genetic diversity, whereas *de novo* variants that arose within a ferret reflected new genetic diversity.

**Fig 5:**
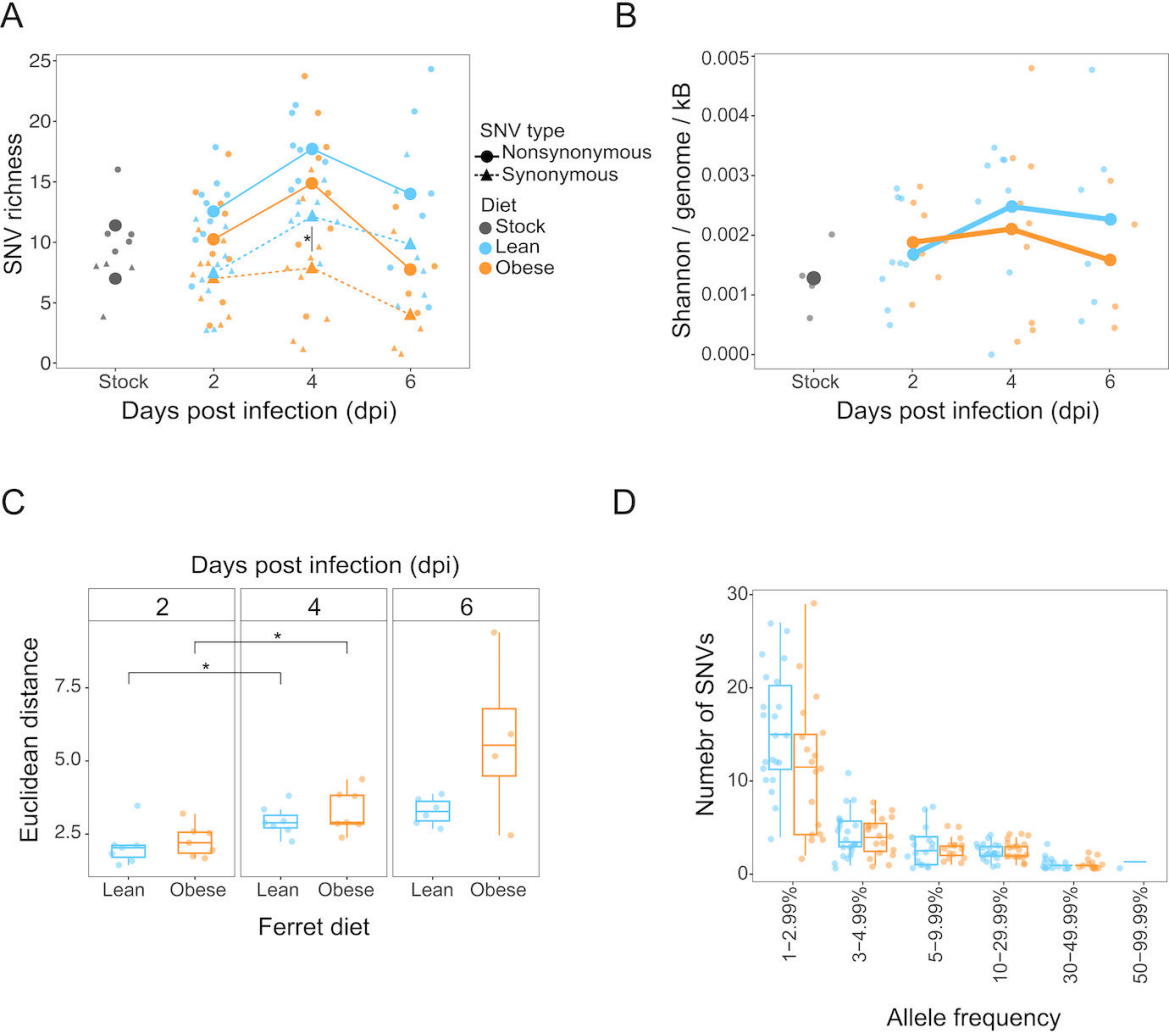
The dynamics of viral genetic diversity did not differ between lean and obese index ferrets. (**A**) Richness per sample calculated by counting the number of SNVs in each index ferret sample. Small points indicate values for individual samples, while larger points connected by lines indicate average values for all samples within a diet group. Color indicates the diet of the ferrets. Dashed lines and triangle points indicate synonymous SNVs while solid lines and circle points indicate nonsynonymous SNVs. All comparisons were not significantly different using two-tailed Student’s *t*-test unless indicated *P < 0.05. (**B**) Shannon entropy per genome per kilobase calculated using the allele frequencies at each SNV position in each index ferret sample. Color indicates the diet of the ferret. (**C**) L2-norm distance (Euclidean distance) calculated between each sample and its associated stock by summing the frequencies of all four nucleotides at all positions within a sample, reflecting all SNVs. Facets indicate dpi and color indicates the diet of the index ferrets. (**D**) Allele frequency distributions of SNVs in index ferrets. Color indicates the diet of ferrets.

We quantified viral genetic diversity using the consensus changes and minor variants in index ferrets (*n* = 12 obese, *n* = 11 lean) using several metrics. First, we calculated SNV richness in each ferret at each time point (Fig. 5A). The richness at 2 dpi was similar to the inoculum and subsequently increased over time, peaking at 4 dpi in both lean and obese index ferrets as a result of the acquisition of *de novo* variants that were not present in the inoculum. Variants were subsequently lost as the infection was cleared and the total viral population decreased as reflected by the decrease in SNV richness detectable at 6 dpi. The overall dynamics were similar for nonsynonymous and synonymous variants and did not differ between diet groups. Comparisons of SNV richness for each segment individually also showed no significant differences between obese and lean ferrets (Fig. S3A). We also calculated diversity using Shannon entropy, which takes into account both the number of variants and their frequencies. This diversity metric showed similar dynamics to SNV richness with a peak at 4 dpi and no significant differences between diet groups (Fig. 5B).

In contrast to SNV richness and Shannon diversity, the genetic distance between nasal wash samples and the inoculum continued to increase over the course of infection (Fig. 5C). This was a result of the simultaneous processes of purging standing genetic variation and introduction of new variation through *de novo* mutations. A substantial fraction of standing genetic variation was maintained in index ferrets over multiple sequential days of infection; however, their frequencies did not differ by more than 10% from their original frequency in the inoculum, suggesting an absence of strong directional selection acting on standing genetic variation (Fig. S3B). At the same time, 46% of variants that were present in the inoculum were not detected at the earliest time point in an index ferret, regardless of diet (Fig. S3C), and most variants were only detected at a single time point (Fig. S3D) indicating a remarkably rapid rate of turnover. The distribution of allele frequencies of these variants was highly skewed to low frequencies (less than 5%), consistent with a high rate of introduction of new mutations (Fig. 5D). Overall, we do not find that the dynamic changes in genetic diversity were impacted by diet.

### DVG diversity is maintained throughout infection

**Fig 6:**
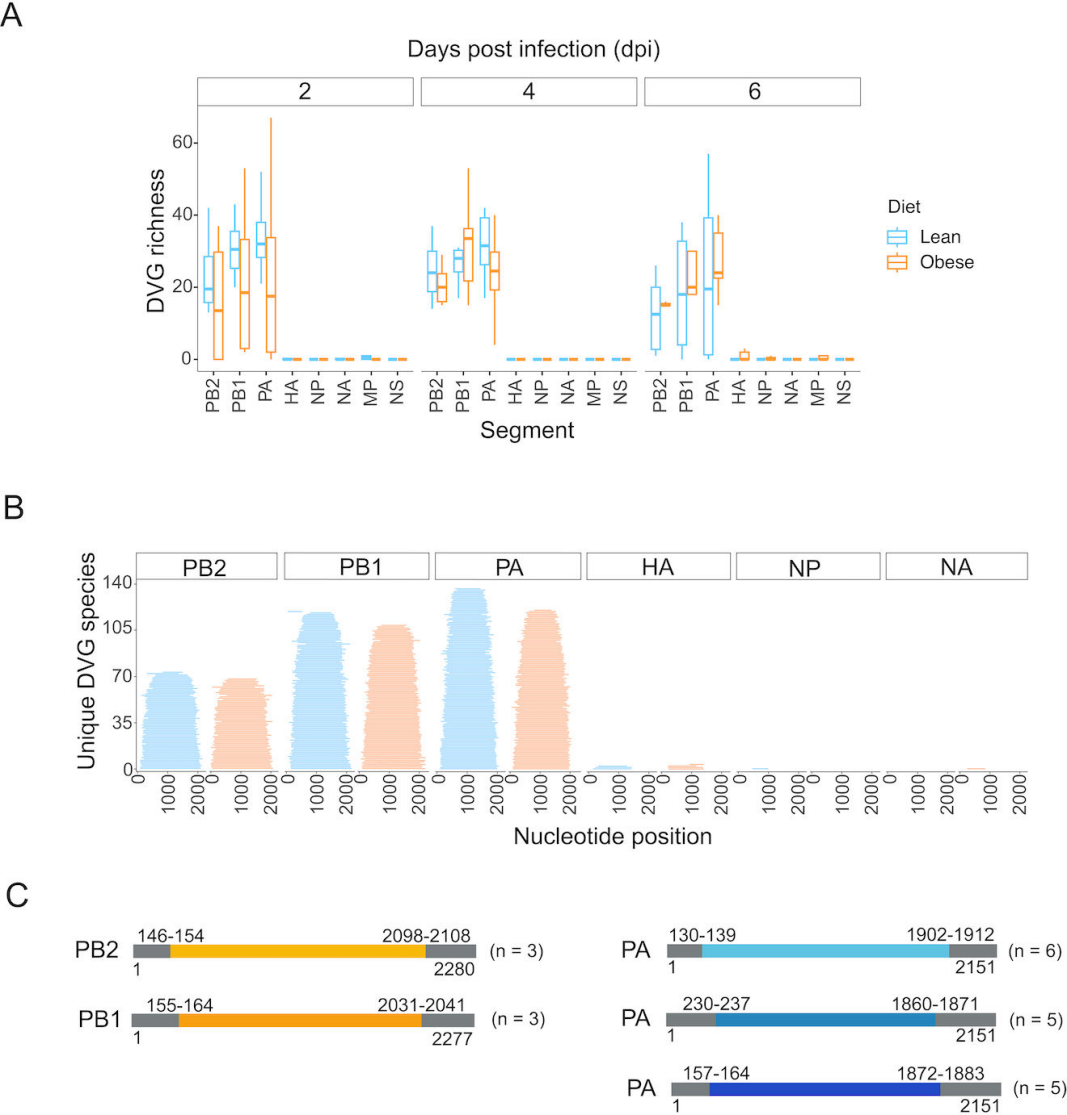
Diversity of defective viral genomes in index ferrets. (**A**) DVG richness per sample calculated by counting the number of unique DVGs in each sample. Facets indicate dpi, and color indicates diet of index ferret. (**B**) Size distribution of the DVGs unique to either obese or lean ferrets. Each line represents the deleted region in a DVG, extending from the start to the end of the deletion. Color indicates diet. Facets indicate segment. (**C**) The most common DVGs within either obese or lean ferrets, with the predicted deletion start and endpoints annotated. The coordinate range of breakpoints is a result of the DiVRGE analysis pipeline grouping DVGs with similar breakpoints. The number of individual ferrets (N) in which the DVG is found is indicated.

Here, we define DVGs as deletions within the gene segments that retain the conserved ends. DVGs have been shown to influence virus-host interactions and to be associated with stronger host immune responses (35). As with SNVs, the diversity of DVGs in contact ferrets may be dependent on both host diet and variation transmitted from the index ferret. To investigate the effect of host diet alone on DVG diversity, we performed DVG detection in index ferret samples using the DiVRGE pipeline (see Materials and Methods). The vast majority of unique DVGs occurred in the three polymerase gene segments (Fig. 6A). Following the initial infection event in index ferrets, DVG richness did not change significantly over the course of infection, regardless of host diet. However, when considering only *de novo* DVGs that were not present in the inoculum, we detected multiple DVGs that arose specifically within a host diet group (Fig. 6B). These DVGs ranged in size from 473 nucleotides to 2,034 nucleotides long—almost the entire length of the PB2 gene—and in general were symmetrical around the segment midpoint with conserved segment ends. There was no significant difference in the size distribution between DVGs that occurred in obese or lean ferrets for any segment. While *de novo* DVGs were typically found in a single ferret, we observed instances in which the same DVG was found recurrently within obese or within lean ferrets (Fig. 6C). The most highly shared DVGs within lean ferrets occurred in the PA segment, whereas the most highly shared DVGs within obese ferrets occurred in the PB2 and PB1 segments, possibly reflecting differential selection pressures as a result of host diet.

## DISCUSSION

In this study, we sought to understand the consequences of host obesity on IAV intrahost evolution. Obesity is associated with significant immune system dysfunction, and therefore, the environmental conditions within the host in which the viral population evolves may be distinct from that of nonobese individuals. However, we did not have an *a priori* expectation about which specific mutations may arise as a result of this selective pressure, as there are likely multiple parallel or complementary ways to increase fitness in an obese environment. To address this question using a controlled experimental approach, we made use of an obese ferret model. We found evidence for specific adaptation of IAV to an obese host through high-frequency variants that were unique to obese individuals. As there are many host factors that could contribute to these dynamics, further functional studies will be needed to confirm the extent to which obesity confers a unique selective environment. By contrast, we did not find that overall IAV genetic diversity differed between obese and lean hosts, highlighting the necessity of considering the specific mutations, their frequencies, and dynamics in the context of host factors. By using an avian-like strain of H9N2 in a ferret model, we also observed signatures of adaptation to the host independent of metabolic state.

Our study design enabled the identification of a unique class of nonsynonymous variants in obese hosts that showed systematic increases in allele frequency over the course of infection and ultimately became the dominant nucleotide. Importantly, we identified this class of variant only in obese contact ferrets. Two of the mutations that exhibited this behavior, PB1 L628M and NP T109I, initially appeared as minor variants in the inoculum, whereas HA S95Y was first identified in the infected contact ferrets but was not transmitted from the index ferret or the inoculum. In both scenarios, the variant only rose to high frequencies after transmission to the contact ferrets.

The unique dynamics observed in obese contact ferrets are likely due to the differences between mechanisms of infection in our experiment. The index ferrets were intranasally inoculated with a very large, genetically diverse viral population. Accordingly, the bottleneck between the stock and the index is wide, with most variants in the stock being found in the index ferrets. However, it is estimated that as few as 2–3 viral particles are passed from index to contact during a transmission event (36, 37). Thus, infection in the contact ferrets was likely seeded by a very small viral population with low genetic diversity, which subsequently expanded and accumulated more variants during the course of infection. We calculated the bottleneck size between the stock and index ferrets and between index and contact ferrets within a transmission pair as described (38). There were 18 stock-to-index ferret events, with a median bottleneck size of 51.5, and 11 index-to-contact ferret events, with a median bottleneck size of 2 (Table S5). These estimates suggest a much tighter bottleneck in index-to-contact transmission than stock-to-index transmission. We also observed that contact samples are more genetically diverged from the stock than index samples (Fig. S1B), consistent with only a small number of variants transmitted between index and contact ferrets. The small founding population size in contact ferrets may allow a single variant to rapidly increase in frequency more easily than in the index ferrets. The comparatively smaller number of lean contact ferrets (*n* = 4) as compared with obese contact ferrets (*n* = 10) was due to the finding that obese ferrets are more likely to transmit and to be susceptible to infection with the H9N2 strain used in this experiment (24). As none of the *de novo* consensus changes are found in more than one ferret, we would not expect to find significant overlap between obese and lean individuals if we increased the number of lean contact ferrets.

PB1 L628M is especially relevant when considering adaptation to the obese host environment as it is a nonsynonymous change that was present in several lean and obese ferrets but only swept to high frequency in obese ferrets. Surprisingly, the PB1 L628M mutation almost always co-occurred with the NP T109I mutation, which may be indicative of an epistatic interaction between these variants (39, 40). During virion production, the viral RNA genome is wrapped around NP proteins and packaged with viral polymerases, which bind to the ends of each of the genomic segments (41). It is possible that the PB1 L628M and NP T109I variants stabilize this complex in a cellular environment with increased inflammatory gene expression, as found in obesity.

In addition to obese-specific consensus changes, there were many minor variants that were unique to either obese or lean ferrets. A total of 12 minor variants were shared between obese ferrets as a result of recurrence. However, these were not shared more frequently than expected by chance and also appear transiently, with most being found at only a single time point. Despite this, we do find that many *de novo* variants are transmitted, which indicates that they may still influence the evolutionary dynamics of IAV. The *de novo* SNVs that we find appear to play a larger role in mediating adaptation to the ferret host, rather than specifically to the obese environment. This reflects the complexity of the environment in which the virus is evolving, in which there is both a strong selective pressure for increased binding and more efficient replication within the ferret host, in addition to the selective pressure created by the physiological and immunological changes in obese hosts.

In addition to SNVs, there were multiple DVGs that were found recurrently within a diet group. DVGs in the PA segment were especially common in the lean ferrets, with several occurring in half, or more, of the lean ferrets. By contrast, even the most highly shared DVGs in obese ferrets occurred in only three individuals (i.e., <30%) and were present on the other polymerase genes (PB1 and PB2). As a DVG needs to infect the same cell as a replication-competent virus in order to be propagated (41, 42), these results suggest that obesity may interfere with the formation and spread of common DVGs and result in less common transient DVGs, perhaps due to the altered cellular environment found in obese ferrets. Previous work has shown that DVGs with similarly sized deletions can have different effects on the interference with the host immune system, suggesting they may be differentially selected, so further functional characterization of these DVGs would be interesting (35).

Although we find evidence for host-specific adaptation, we did not observe differences in the overall dynamics of genetic diversity in obese and lean hosts. In all ferret hosts, we observe a remarkably rapid turnover of diversity as reflected by the ephemeral nature of many SNVs. In index ferrets, diversity peaked 4 days after infection and subsequently declined as the infection was cleared. We also identified several candidate variants that are likely to underlie adaptation to the ferret host regardless of metabolic state as they were present in both lean and obese individuals. Changes in the polymerase gene segments (PB2, PB1, and PA) and the HA segment are associated with viral host switching, and we observed several highly recurrent variants in these genes. Of particular interest is the D685G variant located on the PB1 segment that was found at low frequencies in 22 out of 37 ferrets. Additionally, there were several, highly recurrent variants in the HA1 domain of the HA protein, which may allow more efficient binding to ferret epithelial cells in the respiratory tract, which have different sialic acid distributions than avian species.

Although H9N2 is not currently a seasonal subtype of IAV in humans, it infects avian species and has caused sporadic cases of human disease. As such, it remains a subtype of public health concern. H9N2 has intermediate rates of transmission in the ferret model, which enables assessment of susceptibility to infection through transmission (24). However, the relatively small number of transmission events in our study limited our ability to quantify the diversity of transmitted variants between obese and lean individuals. Nonetheless, our study provides evidence that minor variants are likely transmitted, especially in conditions where direct contact occurs for a period of time, potentially leading to multiple transmission events.

Our study provides evidence that a unique class of variants is selected in obese hosts. However, additional experimentation is required to formally prove that particular variants are uniquely beneficial in obese individuals, as there are potentially complex dynamics influencing their allele frequencies. Future studies will be aimed at assessing the effect of obesity on transmission dynamics and bottleneck sizes. It would also be important to study the extent to which our findings generalize to other IAV subtypes and to extend sampling beyond upper respiratory tracts to study tissue tropism. Finally, integration of host gene expression states may provide mechanistic insights into the molecular basis of differential selective pressures in lean and obese hosts.

## MATERIALS AND METHODS

### Ferret raising protocol

Influenza-naive and neutered male 6-week-old ferrets were obtained from Triple F Farms. Ferrets were randomly assigned to the lean or obese diet groups and fed either a normal or a high-caloric diet for a total of 12 weeks (24). Briefly, lean ferrets were fed a high-density ferret diet once daily in the morning while obese ferrets had free access to a 1:1 mix of high-density ferret diet and feline diet in addition to wet kitten food once per day starting when they were 9 weeks old. Total weight, waist circumference, and skinfold fat measurements were made weekly in order to confirm the obese phenotype.

### Infection experiments and sequencing protocol

A/Hong Kong/1073/1999 virus was propagated in embryonated chicken eggs as previously described (43), and aliquots were stored until use in infection experiments. At this time, they were thawed and diluted in PBS prior to intranasal inoculation of index ferrets with 10^6^ TCID_50_ virus at day 0. On 1 dpi, a naive contact ferret was introduced in the same cage as an index ferret. Ferrets remained co-caged through the end of the experiment at 12 dpi. Experiments were performed using five different cohorts and the results aggregated. Nasal wash samples were collected from anesthetized index and contact ferrets every second day starting at 2 dpi until 12 dpi. Sneezing was induced in anesthetized ferrets (30 mg/kg ketamine delivered intramuscularly, Patterson Veterinary Supply) by instillation of 1 mL PBS supplemented with antibiotics (100 U/mL penicillin and 100 μg/mL streptomycin) to the nasal cavity. Sample was collected, briefly centrifuged, and stored at −80°C. Viral RNA was extracted from 50 µL of nasal wash or the viral inoculum using the QIAGEN QIAmp Viral RNA Mini Kit (QIAGEN, Cat#52904). Viral titers were determined by TCID_50_ analysis.

qPCR analysis was performed using primers targeted against the influenza matrix (M) gene segment: forward 5′-GACCRATCCTGTCACCTCTGAC-3′, reverse 5′-AGGGCATTYTGGACAAAKCGTCTA-3′, probe 5′-TGCAGTCCTCGCTCACTGGGCACG-3′ using the following conditions on a CFX96 Real Time PCR (BioRad): 50°C for 5 minutes and 95°C for 20 seconds, followed by 40 cycles of 95°C for 3 seconds and 60°C for 30 seconds. The copy number of the M gene segment was determined by comparison to a standard curve generated using a FluA gBlock fragment (5′- TCGCGCAGAGACTGGAAAGTGTCTTTGCAGGAAAGAACACAGATCTTGAGGCTCTCATGGAATGGCTAAAGACAAGACCAATCTTGTCACCTCTGACTAAGGGAATTTTAGGATTTGTGTTCACGCTCACCGTGCCCAGTGAGCGAGGACTGCAGCGTAGACGCTTTGTCCAAAATGCCCTAAATGGGAATGGGGACCCGAACAACATGGATAGAGCAGTTAAACTATACAAGAAGCTCAAAAGAGAAATAACGTTCCAT-3′). RNA concentration was measured using a spectrophotometer (NanoDrop).

Viral genomic RNA was amplified using universal Influenza A primers: Uni 12/Inf 1 5′-GGGGGGAGCAAAAGCAGG-3′, Uni12/Inf3 5′-GGGGGGAGCGAAAGCAGG-3′, and Uni13/Inf 1 5′-CGGGTTATTAGTAGAAACAAGG-3′ (IDT). Multiplex reverse transcription-PCR (M-RTPCR) was performed as previously described (44). Amplification was confirmed with gel electrophoresis using a 1% agarose gel stained with ethidium bromide. Samples were diluted to a concentration of 0.2 ng/µL, and sequencing libraries were prepared using the Nextera XT Library Prep protocol (Illumina FC-131-1024). Amplification, library preparation, and sequencing were performed in duplicate for all viral RNA samples (technical replicates). Samples were pooled and sequenced on an Illumina NextSeq 500 using a paired-end (2 × 150 bp) mode (Illumina Inc., San Diego, CA).

### Data processing

Sequence reads were trimmed using Trimmomatic v0.39 with the arguments LEADING:20 TRAILING:20 SLIDINGWINDOW:4:20 MINLEN:20 (45) and aligned to the Influenza A/Hong Kong/1073/1999 (H9N2) reference genome using bwa-mem2 version 2.1 (46). Duplicate reads were marked using Picard v2.23.8 (http://broadinstitute.github.io/picard) and removed using Samtools version 1.11 (47). SNVs were identified using an in-house variant calling pipeline, timo (https://github.com/GhedinLab/timo), using a phred score cutoff of 25. Samples were required to have at least 200× coverage at 40% of positions in all eight genome segments. This ensured that samples with DVGs with low coverage in the middle of the segment were retained. In general, samples had segments with coverage in excess of 1,000×. Forty-percent segment coverage was chosen so that all stock samples were marked as high quality, but samples were manually inspected to confirm their suitability for downstream analysis.

As we performed technical replicates for all samples, we only retained those samples in which both replicates passed threshold cutoffs. Previous work has shown that this leads to a significant decrease in the number of false-positive variants (32). In some cases, there was spurious coverage in one replicate but not the other (although generally, replicates matched each other well) or both replicates failed to pass coverage thresholds; these samples were discarded. For the remaining, high-quality samples, frequency, and coverage values for each variant were averaged using the two replicates. We find that the difference in allele frequency for the vast majority of variants was less than 2% (data not shown). Our experimental design relies on primers targeting the conserved ends of IAV segments, and as a result, we detect variants that fall between these priming sites but not those that would fall within the segment ends. Minor variants were defined as SNVs found between 1% and 50% allele frequency in both replicates (32). Consensus changes were defined as SNVs found at greater than or equal to 50% allele frequency and greater than 10× sequencing coverage in both replicates. Defective viral genomes were identified using DiVRGE (https://github.com/GhedinSGS/DiVRGE) using a minimum deletion size of 5 nucleotides. Downstream analyses were performed in R. All analysis codes can be found on GitHub: https://github.com/GreshamLab/ferret-host-obesity.

### Diversity calculations

Richness was calculated by summing the number of SNVs, regardless of frequency, present in each individual ferret at every time point for which sequencing data were acquired. We calculated richness with respect to the entire genome and each segment.

Shannon entropy was calculated using every SNV, regardless of frequency, for every individual ferret and time point. These values were summed for each sample, divided by the genome size, and normalized to kB by dividing the value by 1000.


Shannon = − Σ (M∗log2(M)) + (A ∗ log2 (A))Normalized Shannon = Shannon / G / 1000

where *M* is the frequency of the major allele, *A* is the frequency of the minor allele, and *G* is the genome size.

Euclidean distance (also known as L2 norm) was calculated using the dist() function in R. Briefly, for every nucleotide position at which there was a variant, the pairwise distance between all samples was determined. The distances for all nucleotide positions were summed to determine the genome-wide distance between samples.


$$Euclidean\;distance\;=\;\sqrt{\Sigma i\;{\left(Xi\;-\;Yi\right)}^{2}}$$

where *i* is the nucleotide position, *Xi* is the frequencies of all four nucleotides at position *i* of sample *X*, and *Yi* is the frequencies of all four nucleotides at position *i* of sample *Y*.

### Neutrality and permutation test

Neutrality tests were performed to analyze if the total number of different shared minor variants found is expected by neutral evolution. Given the average number of different mutations found per ferret (12.5 ± 7.5), we computed the expected distribution of shared and nonshared mutations by simulating the emergence of random mutations in 37 samples (the number of ferrets in our data set). We performed this operation 10,000 times and performed a χ^2^ test to determine the significance of the sharing observed in the experiments.

To test whether minor variants were shared more within a diet group than between diet groups, we performed the following calculations. For any given minor variant that is found *n* number of times, the probability to be found only in lean ferrets and only in obese ferrets is $P_{L}={(N}_{L}/{(N}_{L}+N_{O}){)}^{n}$ and $P_{O}={(N}_{O}/{(N}_{L}+N_{O}){)}^{n}$, where *N_L_* and *N_O_* are the number of lean and obese ferrets, respectively. The probability of being shared among diet groups is $1-P_{L}-P_{O}$ . We computed these probabilities for each of the 100 shared mutations to calculate the expected number of obese and lean specific mutations and the expected number of shared variants, which equals to the sum of probabilities for all of the mutations (i.e., the expected number of obese specific mutations is $\sum_{i=1}^{i=99}P_{Oi}$). We then performed a χ^2^ test comparing the expected number of diet-specific (lean and obese) and shared-among-diet mutations with the observed data.

### Bottleneck calculations

The transmission bottleneck size between (i) stock and index ferret samples and (ii) index and contact ferret samples was estimated based on beta-binomial sampling as described in reference (38). Briefly, the frequencies of all variants found in the first member of the pair, as well as their frequencies in the second member of the pair, were recorded in a table. Variants which were found in the first member of the pair but not detected in the second member were noted at an allele frequency of 0.0 in the second member. Variants only found in the second member of the pair were not included in this analysis, as they could not have been transmitted. For these comparisons, the earliest time point for which positive sequencing data were obtained from each ferret was used, as these most likely represent transmitted rather than *de novo* variants. Once this table was generated for each pair of samples, we used the approximate code (approx.r) with a variant calling threshold of 0.01 to generate an estimate of the bottleneck size between the samples. This code can be found at https://github.com/koellelab/betabinomial_bottleneck. The median of all stock-to-index sample bottleneck size estimates and the median of all index-to-contact sample bottleneck size estimates were then calculated and reported.

## Data Availability

Sequence data have been deposited in the SRA and are available at BioProject ID PRJNA993240.

## SUPPLEMENTAL MATERIAL

File S1 jvi.01778-23-s0001.csv Variant caller output.

Supplemental figures jvi.01778-23-s0002.docx Figures S1 to S3 and all supplemental legends.

Table S1 jvi.01778-23-s0003.xlsx Ferret metadata.

Table S2 jvi.01778-23-s0004.xlsx Transmission pair partners.

Table S3 jvi.01778-23-s0005.xlsx Minor variants in inocula.

Table S4 jvi.01778-23-s0006.xlsx SNVs in all ferrets.

Table S5 jvi.01778-23-s0007.xlsx Bottleneck estimates.

ASM does not own the copyrights to Supplemental Material that may be linked to, or accessed through, an article. The authors have granted ASM a non-exclusive, world-wide license to publish the Supplemental Material files. Please contact the corresponding author directly for reuse.
