# Supplementary material for: Host obesity impacts genetic variation in influenza A viral populations
Source: J Virol. 2024 May 24;98(6):e01778-23. doi: 10.1128/jvi.01778-23 (PMC11237528; doi:10.1128/jvi.01778-23)
Supplement: Supplemental figures — Figures S1 to S3 and all supplemental legends. [file jvi.01778-23-s0002.docx]

# Supplemental Figures

**
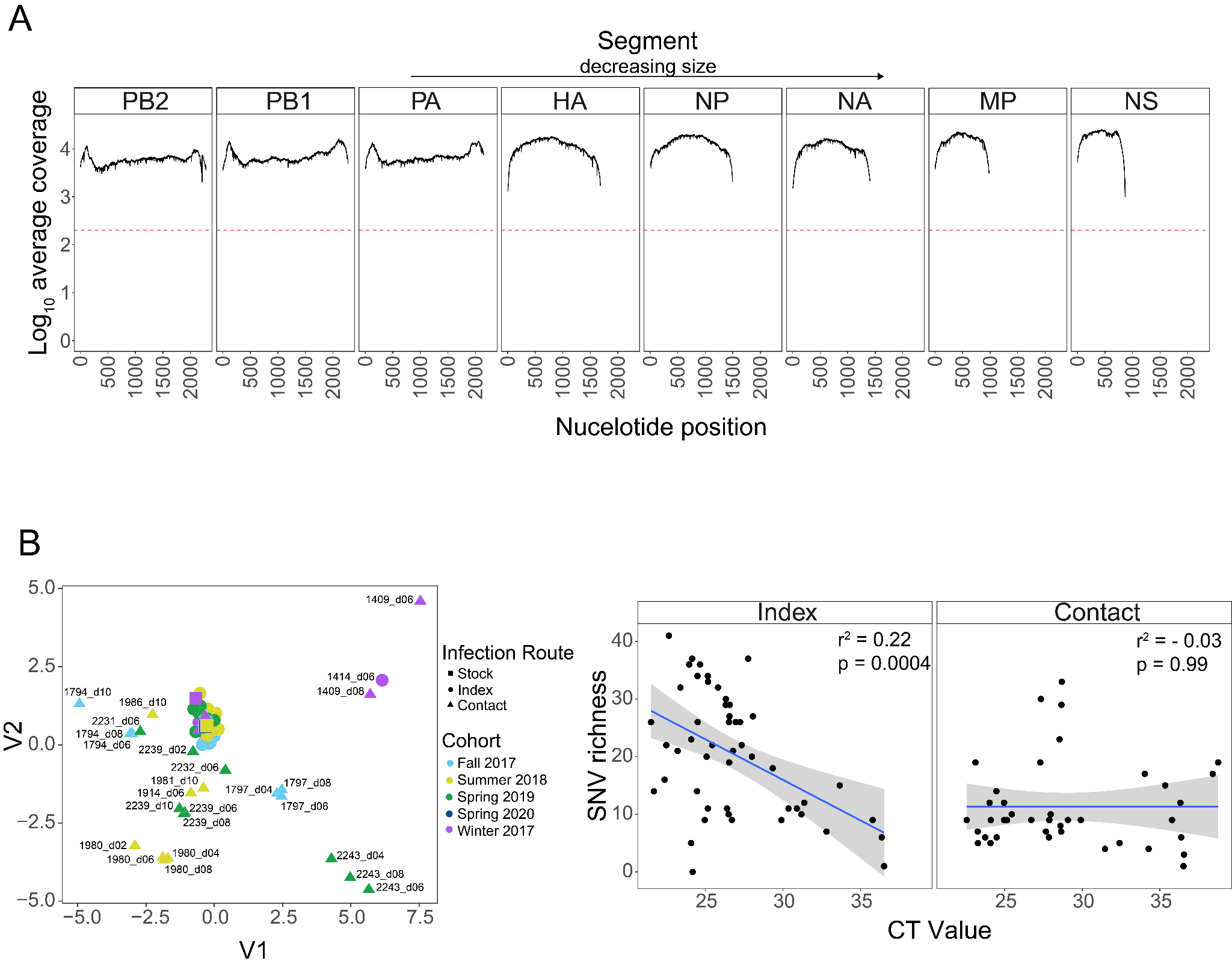
**

**Supplementary Figure 1. Quality control of viral sequencing data. A)** Average coverage across each nucleotide position for all 8 segments of the IAV genome for all samples. Facets indicate segments, which are ordered in decreasing size. **B)** Classical multidimensional scaling of pairwise euclidean distances between all samples. Colors indicate cohort while shape indicates the infection route of that sample. The stock samples cluster in the center, along with most of the index samples, while the contact samples are more dispersed, and therefore more genetically different from the other samples. **C)** Correlation between qPCR Ct values and number of SNVs for each sample. A linear model was fit to the data and the Pearson correlation (r^2^) and associated p-value determined.


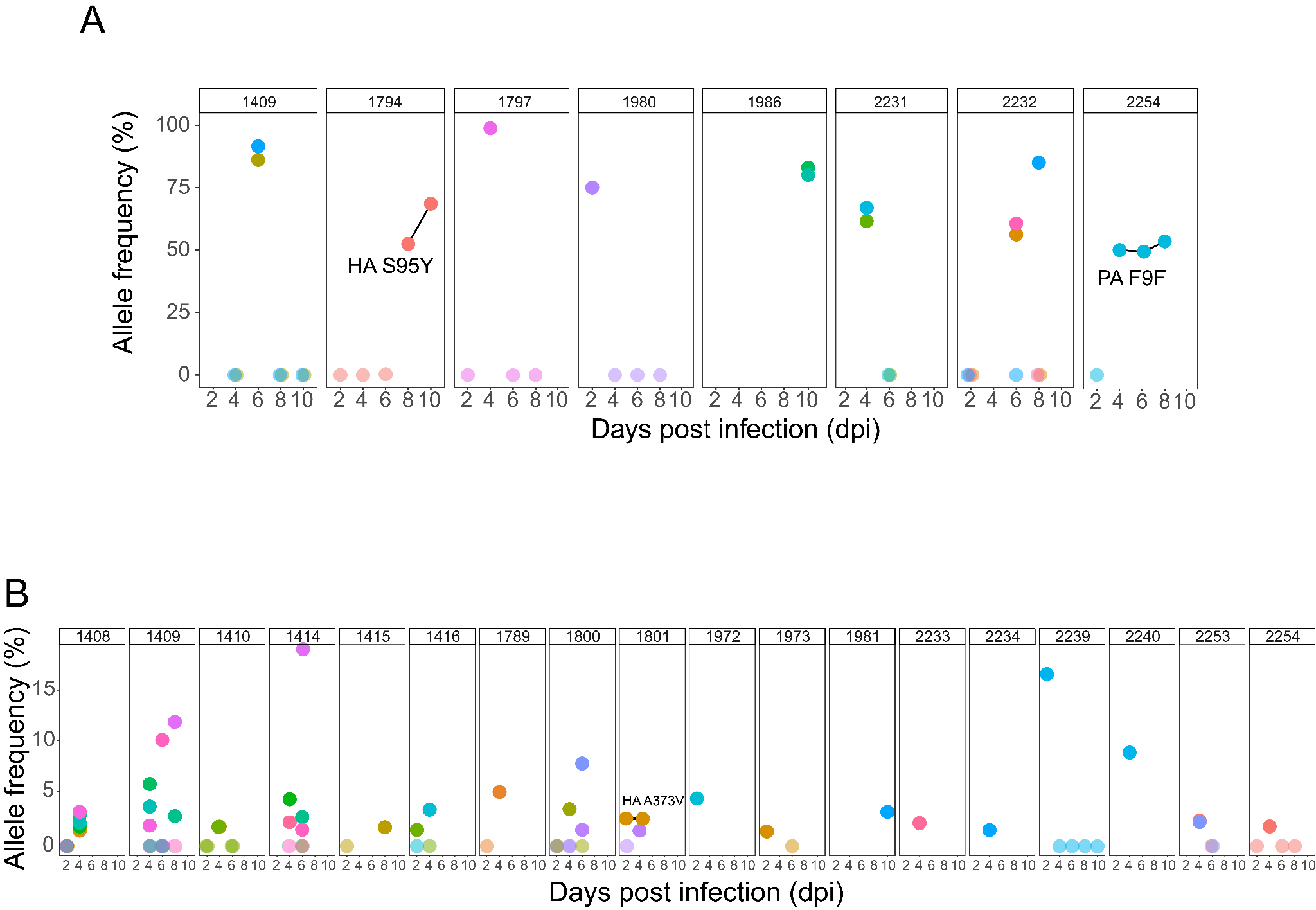


**Supplementary Figure 2. Persistence of consensus changes and minor variants. A)** Allele frequency of all consensus changes. Lines connecting points indicate persistence of a variant for at least two time points. Persistent variants are labeled with their amino acid change. **B)** Allele frequency of recurrent *de novo* nonsynonymous minor variants identified in obese ferrets. Line connecting points indicate persistence of a variant for at least two time points. Persistent variants are labeled with their amino acid change. For both panels, points at AF = 0 indicate a sample where there was high quality sequencing data but the variant was not detected.


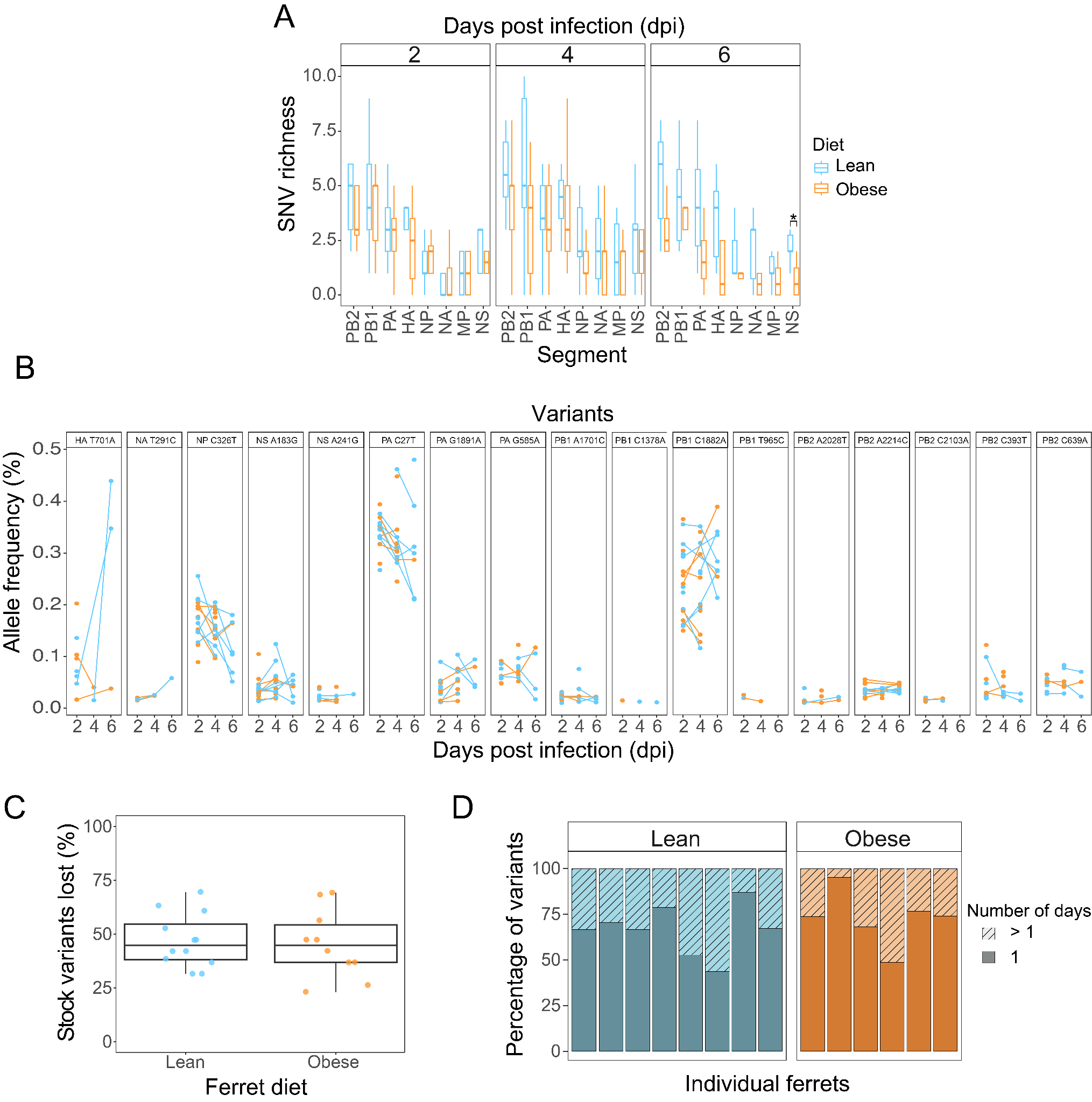


**Supplementary Figure 3. SNV dynamics in index ferrets. A)** Distribution of SNV richness for each genome segment over all samples for days 2-6 dpi. **B)** Turnover of minor variants in index ferrets. Facets and color indicate diet of index ferret **C)** Percentage of minor variants present in the stock that was identified in each index ferret. **D)** Variants that were identified at only one time point or at multiple time points. Shading indicates whether a variant was found in only a single time point (dark) or multiple time points (light and striped) for the same ferret.

#

# Supplementary Tables

**Table S1.** Metadata for all ferrets included in the study with physiological measurements taken prior to infection.

**Table S2.** Transmission pair partners.

**Table S3.** SNVs identified in each inoculum, their amino acid change, frequency, and the number of inocula in which they were found.

**Table S4.** SNVs identified in each ferret, their amino acid change, and frequency.

Supplementary Files

**Supplementary File 1.** Final output of the timo variant calling pipeline used for all downstream analyses performed in R.
